# Clock-talk: have we forgotten about geographic variation?

**DOI:** 10.1007/s00359-023-01643-9

**Published:** 2023-06-16

**Authors:** William E. Bradshaw, Margaret C. Fletcher, Christina M. Holzapfel

**Affiliations:** Laboratory of Evolutionary Genetics, Institute of Ecology and Evolution, University of Oregon, Eugene, OR 97403-5289 USA

**Keywords:** Evolution, Genetic correlation, Phylogeography, Micro-evolution and macro-evolution, Species diversification, Photoperiodism

## Abstract

*Wyeomyia smithii*, the pitcher-plant mosquito, has evolved from south to north and from low to high elevations in eastern North America. Along this seasonal gradient, critical photoperiod has increased while apparent involvement of the circadian clock has declined in concert with the evolutionary divergence of populations. Response to classical experiments used to test for a circadian basis of photoperiodism varies as much within and among populations of *W*. *smithii* as have been found in the majority of all other insects and mites. The micro-evolutionary processes revealed within and among populations of *W. smithii*, programmed by a complex underlying genetic architecture, illustrate a gateway to the macro-evolutionary divergence of biological timing among species and higher taxa in general.

**Supplementary Information:**

The online version contains supplementary material available at 10.1007/s00359-023-01643-9.

## Introduction

The great rhythms of the biosphere in light, temperature, and moisture are due to the daily rotation of the earth around its own axis and by the annual orbit of the earth around the sun. The tilt of the earth in its elliptical orbit is responsible for the changing seasons and for the opposition in the progression of the seasons in the southern and northern hemispheres. These rhythmic environments impose selection for temporal programming by organisms living on planet Earth. Daily circadian clocks are ubiquitous from Cyanobacteria to humans, while the annual change in day length (photoperiod) orchestrates the delicate balance of seasonal timing from rotifers to rodents. Photoperiod provides a highly reliable anticipatory cue for future seasonal change in the environment, but only if there is an annual fluctuation in day length (Danilevskii 1965, p. 34). Although photoperiodic insects occur within 9° latitude of the Equator (Norris and Richards 1965; Tanaka et al. 1988), it is among denizens of the temperate and subarctic zones above 30° latitude where there is a strong day-length signal that photoperiodism is most prevalent (Taylor and Spalding 1986, Fig. 5.2a; Bradshaw and Holzapfel 2007; Hut et al. 2013, Fig. 3). The original invaders of higher latitudes would already have possessed circadian clockworks before strong local selection was imposed on photoperiodic timing in the new seasonal environment (Hut and Beersma 2011).

After the initial experimental demonstration of the importance of day length for seasonal flowering in a variety of agriculturally important plants (Garner and Allard 1920), Erwin Bünning (1936, p. 590) proposed that the measurement of day length is one of the functions of the circadian clock.^1^ A great deal of research has been invested in testing Bünning’s hypothesis, but much hinges on the meaning of *Grundlage*. In his own words (Bünning 1973, p. 223), he said that “…it is no longer surprising to us that certain developmental processes may or may not be coupled to the clock.” Bünning’s use of “coupled” implies three functional entities: the circadian clock, the photoperiodic timer, and a connection between them. Herein, we explore the physiological and evolutionary connection between the circadian clock and the photoperiodic timer in the pitcher-plant mosquito, *Wyeomyia smithii*. We discuss our discoveries in the contexts of not only the clock-timer relationship, but also how variation in this relationship within *W. smithii* provides a gateway between micro- and macro-evolutionary processes of biological timing.

### External coincidence

Bünning’s (1936) hypothesis was based on a night-long sensitivity to light. Pittendrigh and Minis (1964) developed the external coincidence model as a refinement of Bünning’s hypothesis. They proposed that light had a dual function: setting the phase of an underlying narrow circadian sensitivity to light and then triggering a long-day response if external light coincided with “photoperiodically inductive coincidence” of the rhythm (Pittendrigh and Minis 1964, Fig. 14), later, and since, known as the “inducible phase” or φ_i_ (Pittendrigh 1966). Photoperiodic response in *Wyeomyia smithii* is consistent with the external coincidence model (Emerson et al. 2008, 2009).

### Experimental protocols

Two main experimental protocols are used to test for a connection between the circadian clock and the photoperiodic timer: resonance and light-break experiments.

**Fig. 1:**
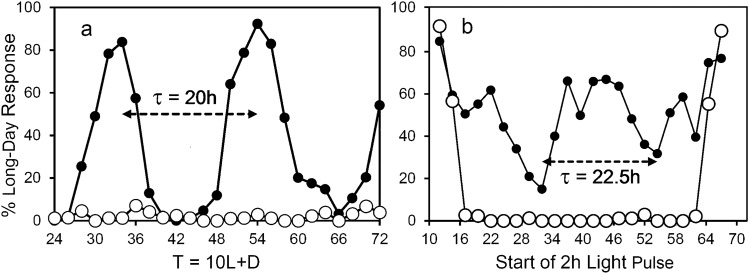
Positive (filled circle) and negative (open circle) responses to **a**, resonance experiments and **b**, interrupted night experiments. Peak-to-peak or valley-to-valley interval serves as an indicator of the period (*τ*) of the circadian oscillator connected with the photoperiodic timer

Resonance experiments are also known as the Nanda-Hamner protocol after Nanda and Hamner (1958) or “T” experiments because treatments consist of varying the length of the extrinsic L + D = T cycles. In resonance experiments, a fixed short day is followed in separate treatments by varying night lengths to create light + dark = 24–72 h, usually by 1 h or 2 h increments. When the T cycle resonates with the underlying circadian clock, a short-day response is maintained; when the T cycle is discordant with the underlying circadian clock, light falls in the sensitive phase of the cycle and a long-day response ensues. When long-day response is plotted against T = 24–72 h, a “positive” result is indicated by a biphasic long-day response with a peak-to-peak interval reflecting a circadian period of ~ 24 h (Fig. 1a; Nanda and Hamner 1958; Pittendrigh 1981; Vaz Nunes et al. 1990; Teets and Meuti 2021).

Light-break experiments are also known as the Bünsow protocol (1960) or asymmetric skeleton photoperiod experiments (hereafter, ASPP). As illustrated by Pittendrigh and Minis (1964, Fig. 16), the long night of an otherwise short day is scanned in separate experiments by a succession of brief light pulses. Usually, this protocol elicits two discrete peaks of long-day response: an early night “A” peak and a late night “B” peak. The A peak serves as a delayed lights-off and the B peak as an advanced lights-on (Pittendrigh and Minis1964). Results of short-day plus long night cycles of 24 h or 48 h can reveal both consistent and discordant responses to ASPPs among species, populations, or selected lines. A “positive” result is indicated only by a mid-dark, long-day response in 72 h cycles, with a valley-to-valley interval reflecting a circadian period of ~ 24 h (Fig. 1b; Saunders1970; Saunders et al. 2004).

### Wyeomyia smithii

**Fig. 2:**
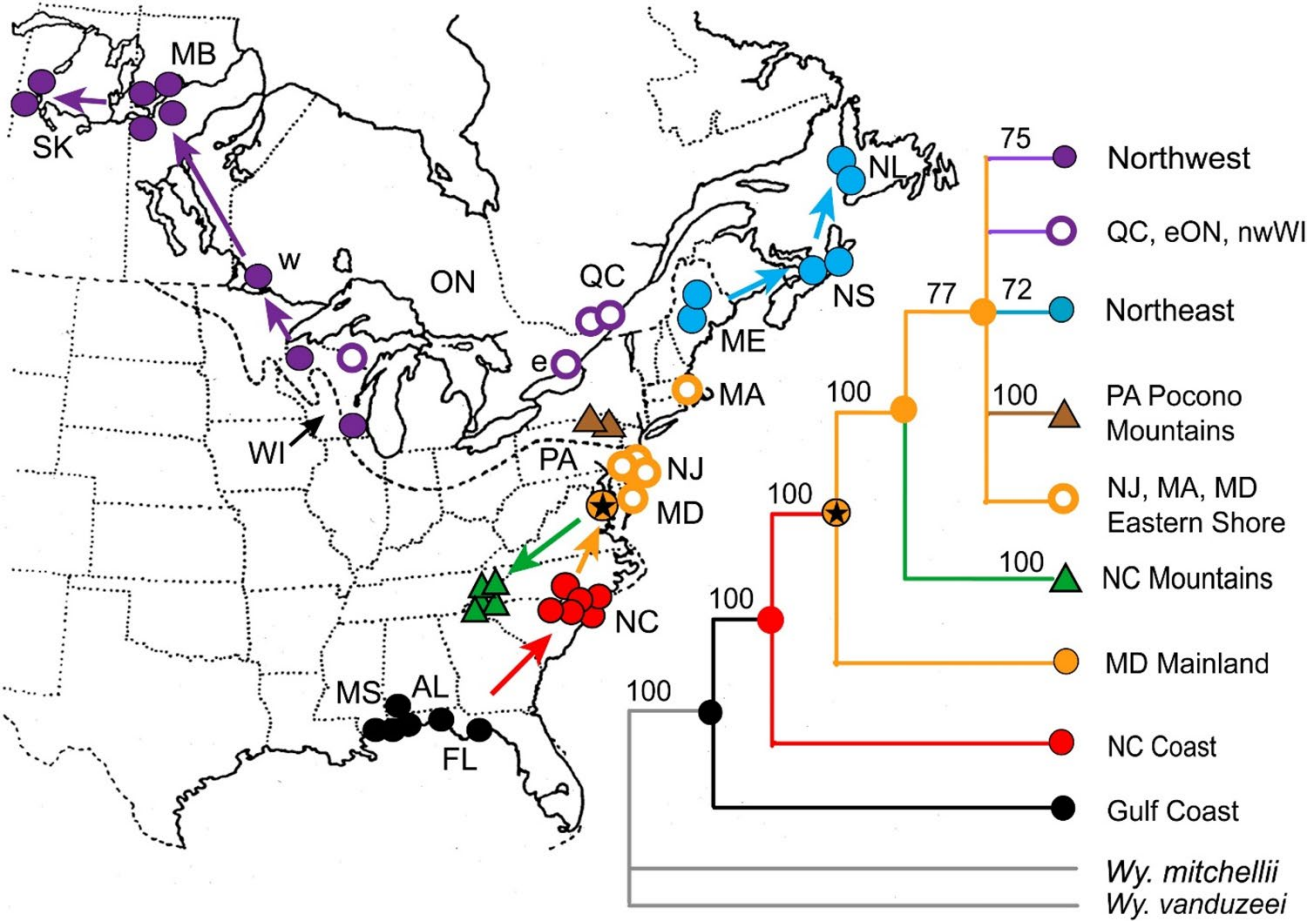
Phylogenetic relationship among populations. Ancestral *Wyeomyia smithii* dispersed from the Gulf Coast, north along the eastern coastal plain and Piedmont Plateau, then branched into three separate major clades to the northeast, the northwest, and the southern Appalachians. Mountain populations are distinguished by triangles. Colored arrows show direction in evolution. From Merz et al. (2013)

Pre-adult *Wyeomyia smithii* are obligate inhabitants of the carnivorous pitcher plant, *Sarracenia purpurea*. Ancestral *W. smithii* originated along the Gulf of Mexico and migrated along the eastern coastal plain and Piedmont Plateau to the Mid-Atlantic states, thence post-glacially northeast to Newfoundland, northwest to at least Saskatchewan, and southward in the Appalachian Mountains (Bradshaw and Lounibos 1977; Fig. 2). Hence, the geographic distribution of *W. smithii* across both latitudinal and altitudinal gradients represents the current end points of their evolutionary history. There are two major clades, a southern clade including populations ranging from the Gulf Coast to the North Carolina coast and Piedmont Plateau, and a northern clade including all more northern and mountain populations (Bradshaw and Lounibos 1977; Merz et al. 2013). Herein, we focus on six clades, two southern Gulf Coast, coastal and piedmont North Carolina (NC Coast), and four northern, mid-Atlantic (mid-latitude), northeast, northwest, and mountain, the latter three sharing a most recent common ancestor with the mid-latitude populations (Fig. 2).

Over this range, *W. smithii* diapause as larvae and overwinter in the water-filled leaves of their host plant (Bradshaw and Lounibos 1977). Larval diapause is initiated and maintained by short days and averted or terminated by long days (Smith and Brust 1971; Bradshaw and Lounibos 1972, 1977). The critical photoperiod regulating diapause increases predictably with both latitude and altitude (Bradshaw 1976; Bradshaw and Lounibos 1977; Bradshaw and Holzapfel 2001a; Bradshaw et al. 2003). Response to resonance experiments shows a strong biphasic response in southern populations, expected from a coupling between photoperiodism and the circadian clock, with an estimated period of oscillation of 20–21 h. The amplitude, but not the period of the apparent oscillation declined in progressively more derived populations, becoming totally non-responsive in the NC mountain populations (Bradshaw et al. 2003, 2006). Divergent selection on critical photoperiod in three subpopulations at mid-latitude resulted in divergent photoperiodic response of critical photoperiod. In addition, there was a correlated response to 10 week long resonance experiments: the amplitude increased in short-selected lines (more southern like) and decreased in long-selected lines (more northern like). These replicated results show that there was individual genetic variation within each sub-population for response to resonance experiments and a negative genetic correlation between critical photoperiod and amplitude of response to resonance experiments (Box 1). Antagonistic selection against the genetic correlation reverses the sign of the correlation from negative to positive in five cycles of selection (Bradshaw et al. 2012). If one accepts a rhythmic response to resonance experiments as a proxy for the circadian clock, these results mean that (1) there was sufficient time for the photoperiodic counter to sum to a developmental response, and (2) the amplitude of the circadian pacemaker and/or its connection to the photoperiodic timer is genetically variable and has a declining influence on photoperiodic response in progressively more recently derived populations.

Finally, crosses between the selected lines within a population and between divergent populations reveal not only that the independent effects of alleles (additive genetic variance) but also that allelic interactions (dominance) and gene–gene interactions (digenic epistasis) contribute to standing genetic variation in critical photoperiod (Hard et al. 1992, 1993; Lair et al. 1997; Bradshaw et al. 2005) and response to resonance experiments (Mathias et al. 2006).

Herein, we explore geographic variation in response to ASPPs, compare results from ASPPs with response to resonance experiments and with results from numerous other species. We discuss clock-timer connectivity and how micro-evolutionary processes seen within *W. smithii* provide the template for elucidating macro-evolutionary patterns among species of insects and mites. We conclude that phenotypic and genetic variation within *W. smithii* alone serves as a micro-evolutionary example that, if mirrored among populations within other species, would account for macro-evolution of photoperiodic timing.

Box 1. Correlated response to selection (Falconer 1981, Eq. 19.6). ${\mathrm{CR}}_{Y} = \frac{{S}_{X}}{{\delta }_{PX}}\left({h}_{X}{h}_{Y}{r}_{A}{\delta }_{PY}\right),$

CR_Y_ = correlated response in trait Y to direct selection on trait X.

S_X_ = selection differential imposed on trait X.

δ_PX_ = phenotypic standard deviation in trait X.

δ_PY_ = phenotypic standard deviation in trait Y.

h_X_ = √(heritability of trait X).

h_Y_ = √(heritability of trait Y).

r_A_ = additive genetic correlation between trait X and trait Y.

For there to be a correlated response, each of the elements in the parentheses must be non-zero.

## Materials and methods

### Collection and maintenance

We collected larvae of *Wyeomyia smithii* during the overwintering generation from 16 localities in eastern North America (Table 1). Populations can be geographically grouped as southern (AL & FL, 30–31°N), lowland North Carolina (NC, 34–35°N), mountain North Carolina (NC, 35–36°N, 900 m elev), intermediate (MD, NJ & PA, 38–42°N), northeastern (ME, 46°N), and northwestern (WI & ON, 46 & 49°N). At least 2000 larvae were collected from each locality. All populations were raised for at least three generations prior to the start of experiments to reduce field effects. Experimental animals were systematically sampled from a continually reproducing stock population. Stock dishes of 35 larvae each were organized by oviposition date and every *n*th dish removed, pooled, and re-allocated to previously labeled, haphazardly scrambled experimental dishes. Once removed from stock, no experimental larvae were ever returned to stock. Larvae used in experiments were reared on short days (L:D 8:16) at 21 ± 0.5 °C for at least 30 days before the start of an experiment to synchronize development and to ensure that they were in diapause.

**Table 1:** Population origins, acronyms, critical photoperiods, and symbols in figures

Origin^a^	POP	°N LAT	°W LON	M ELEV	CPP	Symbol
NW, ON	DL	50	50	410	15.4	●
NW, WI	ML	46	90	500	15.0	◯
NE, ME	KC	46	68	370	15.4	●
MID, PA	TH	41	75	600	14.4	▲
MID, NJ	HV	40	75	10	13.4	
MID, NJ	MM	40	75	10	13.4	X
MID, NJ	PB	40	74	10	13.5	◯
MID, MD	NP	38	75	20	13.4	●
NC MTN	CB	36	83	900	14.0	
NC MTN	DB	35	83	900	13.9	▲
NC MTN	HK	35	83	900	13.9	△
NC COAST	PM	35	80	90	13.0	●
NC COAST	GS	34	78	20	12.7	◯
GULF COAST, FL	CR	31	87	40	12.4	
GULF COAST, AL	LI	30	87	15	12.3	◯
GULF COAST, FL	WI	30	85	10	12.1	●

**Fig. 3:**
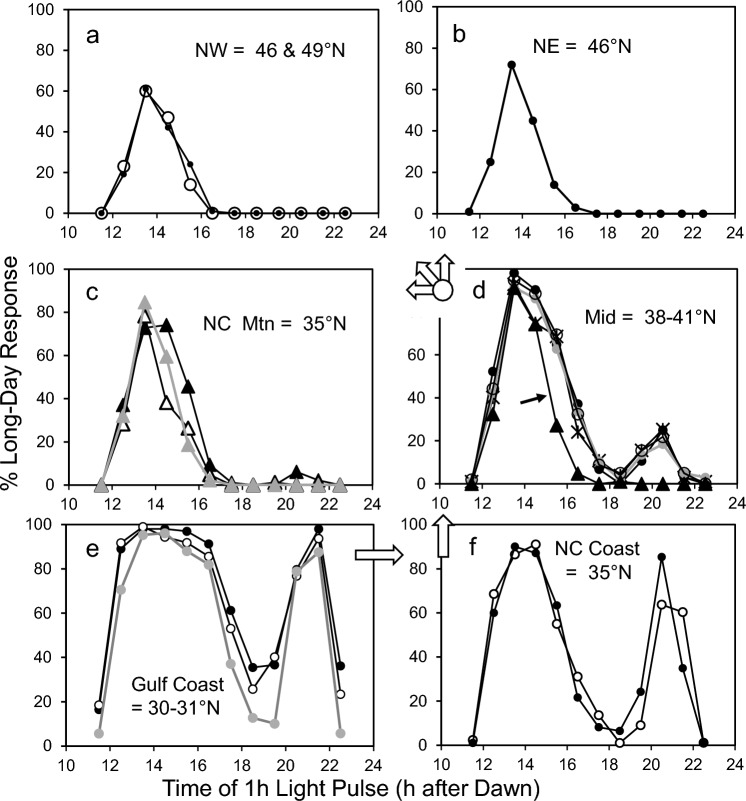
Developmental (long-day) response of diapausing *W*. *smithii* to 1 h light pulses during the night of an otherwise diapause-maintaining short-day photoperiod with a 14 h-long night (L:D = 10:14). Arrows show direction in evolution (Fig. 2) from the Gulf Coast (**e**), north along the eastern coastal plain and Piedmont Plateau (**f**, **d**), then branching into three separate clades to the northeast (**b**), the northwest (**a**), and the southern Appalachians (**c**). Symbols as in Table 1. Mountain populations are distinguished by triangles; the small arrow inset in d indicates the population from the Pocono Mountains in Pennsylvania

**Fig. 4:**
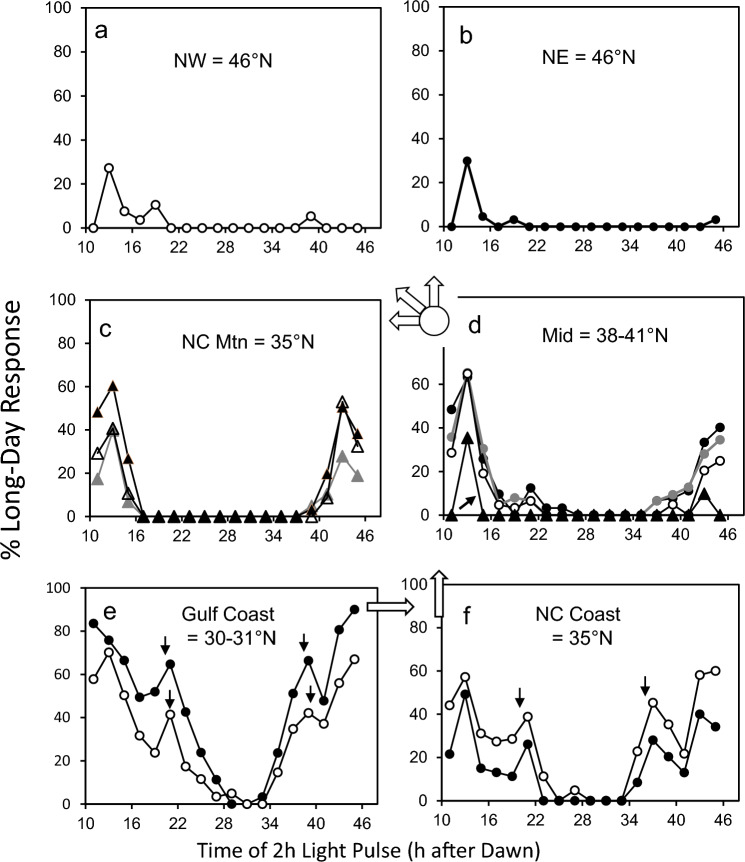
Developmental (long-day) response of diapausing *W*. *smithii* to 2 h light pulses during the night of an otherwise diapause-maintaining short-day photoperiod with a 38 h-long night (L:D = 10:38). Symbols as in Fig. 3

**Fig. 5:**
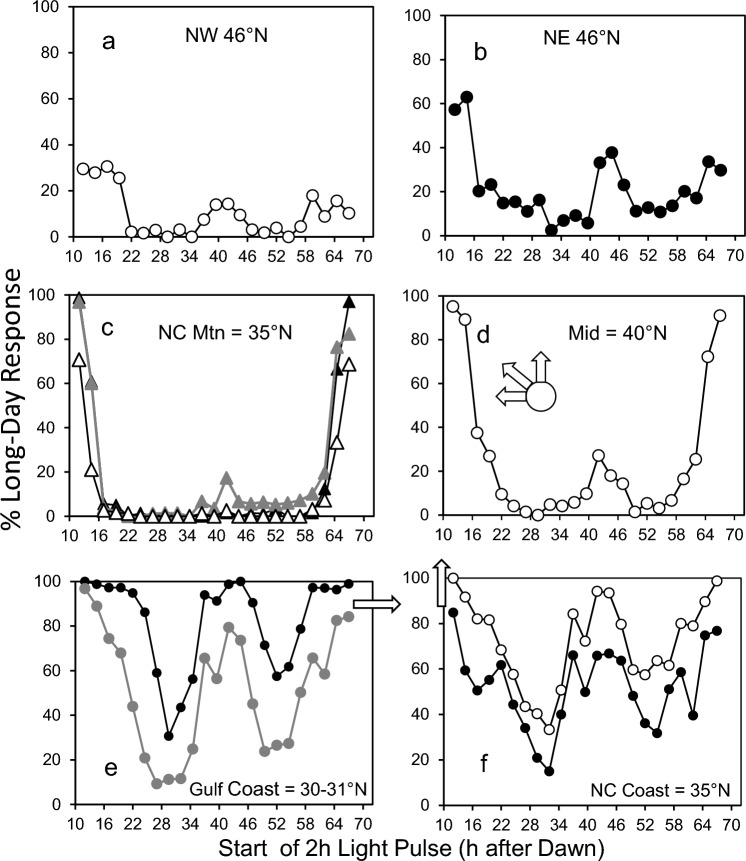
Developmental (long-day) response of diapausing *W*. *smithii* to 2 h light pulses during the night of an otherwise diapause-maintaining short-day photoperiod with a 62 h-long night (L:D = 10:62). Symbols as in Fig. 3

*Pop* population-specific acronym used in previous publications from this lab; *Latitude* Longitude & Elevation of origin; *CPP* critical photoperiods from Bradshaw et al. (2003) and Wegis et al. (1997); symbols as used in Figs. 3, 4, 5

^a^Origin, region and state or province where collected

### Experimental conditions

Experiments were carried out in a controlled-environment room at 23 ± 0.5 °C inside light-tight photoperiod cabinets where larvae were exposed to a 4W, cool-white fluorescent lamp at a distance of 10–20 cm. To avoid a parallel thermoperiod, the ballasts were located outside the cabinet and each bulb was housed in a clear polycarbonate tube through which 100ft^3^/min forced air was blown. Experimental L:D cycles were controlled by Chrontrol^®^ electronic timers. At the beginning of the experiment and twice a week thereafter, the dishes of larvae from each of the sixteen populations were haphazardly arranged within each photoperiod cabinet without regard to latitude or altitude of the population. Experimental animals were fed a 3:1 by volume a mixture of ground Geisler^®^ guinea pig chow and San Francisco Bay Brand® brine shrimp. They were fed, cleaned, and checked for pupae, which were removed twice a week for the duration of the experiment.

### Experimental treatments

Larvae were exposed to the experimental treatments for 30d (*T* = 24, 48) or 56d (*T* = 72) at 21 ± 0.5 °C. After the 30 or 56 days, larvae were transferred to short days (L:D 8:16) at 21 ± 0.5 °C for two additional weeks to allow and record development of larvae that had been stimulated by the L:D cycle during the experiment but that had not pupated by the 30th or 56th day. At the end of the two weeks in short day, remaining larvae were censused and then discarded. Percentage development was calculated for each replicate as (100) × (total number of larvae having pupated) ÷ (total number of larvae having pupated + number of larvae remaining alive on day 44 or 70).

To determine response to light breaks during an otherwise short day and long night, we exposed larvae to a 10 h, diapause-maintaining day length combined with a 14 h (*T* = 24), 38 h (*T* = 48), or 62 h (*T* = 72) night length. Beginning one hour after lights off, the night was systematically scanned with a 1 h light pulse in 12 separate experiments when *T* = 24, with a 2 h light pulse in 18 separate experiments when *T* = 48, and with a 2 h light pulse in 23 separate experiments when *T* = 72. Not all populations were used in each T-cycle experiment, but each T-cycle experiment (*T* = 24, 48, or 72 h) was carried out as a single block with all populations experiencing a given treatment concurrently in the same cabinet. We knew a priori from previous experiments (Bradshaw et al. 1998) that 30 days were sufficient to elicit a long-day response with 24 h and 48 h cycles. To make sure we imposed sufficient cycles to elicit a long-day response for *T* = 72, we included a long-day control with L:D = 18:54. For *T* = 72, we included only 10 populations due to the logistics of randomizing and setting up concurrent treatments (24,000 larvae) while retaining 5000 additional larvae from each population as stock. For each experimental cycle (*T* = 24, 48, or 72) larvae were randomized, allocated to experimental treatments as a single block with all populations experiencing a given light break treatment doing so in the same individual photoperiod cabinet. For each experimental cycle, experiments were initiated on the same date.

Graphics were plotted using Excel and PowerPoint in Microsoft Office 2019.

## Results

### 24 h cycle

Figure 3 shows the developmental response of diapausing larvae to a 10:14 = L:D cycle with 1-h light pulses during the 14-h dark period. Southern populations along the Gulf Coast show robust A and B peaks. The A peak declines in breadth and amplitude in progressively more derived populations. The B peak also declines in progressively more derived populations and is absent entirely in the Pennsylvania mountain population (Fig. 3d, arrow), in two of the three North Carolina Mountain populations (Fig. 3c), and in both northeast and northwest populations (Fig. 3a, b).

### 48 h cycle

Figure 4 shows the developmental response of diapausing larvae to a 10:38 = L:D cycle with 2 h light pulses during the 38 h dark period. The A and B peaks occurred in the early and late subjective night in the Gulf Coast, NC Coast, NC Mountain, and Mid-Latitude populations. The breadth and amplitude of the peaks declined in progressively more derived populations, with the B peak essentially absent in the northern populations. In both the Gulf Coast and NC Coast populations, there appeared secondary “C” peaks in the early-mid and late-mid subjective nights; both C peaks were absent in more derived populations.

### 72 h cycle

Figure 5 shows the developmental response of diapausing larvae to a 10:62 = L:D cycle with 2-h light pulses during the 62-h dark period. Response to long-day controls (L:D = 18:54) ranged from 97–100%. Both A and B peaks were discernable in the early and late subjective nights, declining in breadth and amplitude in more derived populations. The “C” peaks were reliably discernable in only one NC Coast population (Fig. 5f●). A broad peak in the middle of the subjective night was prominent in the Gulf Coast and NC Coast populations, declining but discernable in more derived populations.

## Discussion

“It is not even clear that the diversity of individual photoperiodic responses within the same insect are all based on the same strategy for measuring photoperiod” (Pittendrigh 1984, p. 37).

### Treatments

The experiments in Figs. 3, 4, 5 were each run as a single block, with all populations experiencing a given ASPP treatment within the same experimental chamber. Hence, variation among populations in response to a given ASPP reflects variation of populations experiencing the same photic environment at the same time. In addition, each plot in Figs. 3, 4, 5 represents the results of two or more populations, not replicates from a single population. The exceptions are northeast vs. northwest comparisons, for which we have only a single population (Figs. 3b, 4, 5a, b). Consequently, we discuss them collectively as “northern” or “more highly derived,” for which they become replicate populations separated by > 25° longitude.

### 24 h and 48 h cycles (*T* = 24 & 48)

The most apparent pattern in both 24 h and 48 h cycles is a decreasing long-day response with increasingly derived more northern and high-elevation populations. When *T* = 24, southern populations exhibited the expected biphasic response that declined to a single A peak in the early subjective night in northern populations. When *T* = 48, southern populations exhibited A, B, and two “C” peaks, declining to a single, relict A peak in the northern populations. Like long-day response the complexity of response also declined with increasing descent. Most of the variation in response to ASPPs among different species in seven orders of insects and mites is represented within *W. smithii* alone (Table 2). Pittendrigh’s (1984, p. 37) “diversity of individual photoperiodic responses within the same insect” is well illustrated by responses to 24 h and 48 h T cycles in *Wyeomyia smithii*.

**Table 2:** Patterns of response to interrupted-night experiments (ASPP) in *Wyeomyia* and other insects and in mites

Species	Pop,°N	T, P, L	*Wy *(Notes)	References
*Aedes atropalpus*	1p	24, 30, 10	(3)	Beach and Craig (1977, Fig. 3b)
*Aphis fabiae*	1p	24, 60, 10	NC Cos	Hardie (1987, Fig 3)
*Chaoborus americaus*	1p	24, 30, 8	NC Cos (6)	Bradshaw (1974, Fig. 3)
*Chilo suppressalis*	1p	24, 60, 12	(12)	Xiao et al. (2010, Fig. 4)
*Colaphellus bowringi*	1p	24, 120, 12	(12)	Wang et al. (2004, Fig. 3)
*Drosophila auraria*	2p,9°	24, 60, 10	NC Cos (3)	Pittendrigh and Takamura (1993, Fig. 2)
*Drosophila triauraria*	2p,2°	24, 60, 10	(13)	Yoshida and Kimura (1994, Fig 4B)
*Mamestra brassicae*	1p	24, 60, 10	(10)	Masaki (2001, Fig 3C, D)
*Megoura viciae*	1p	24, 60, 13.5	NW NE NC Mtn	Lees (1973, Fig. 2D)
*Megoura viciae*	1p	24, 60, 12	NC Mtn	Zaslavski (1992, Fig. 2)
*Metriocnemus knabi*	1p	24, 90, 12	(6)	Paris and Jenner (1959, Table VI)
*Nasonia vitripennis*	1p	24, 60, 12	NC Cos	Saunders (1970, Fig. 3A)
*Ostrina furnacalis*	1p	24, 60, 9	(6)	Yang et al. (2014, Fig. 4B)
*Ostrinia nubilalis*	1p	24, 60, 7	(3)	Beck (1976, Fig. 3)
*Ostrinia nubilalis*	1p	24, 60, 12	Mid	Skopik et al. (1986, Fig. 2)
*Ostrinia nubilalis*	1p	24, 120, 12	(5)	Bonnemaison (1978, Fig. 5)
*Pectinophora gossypiela*	1p	24, 60, 10	NC Cos	Adkisson (1966, Fig. 4)
*Pieris brassicae*	1p	24, 30, 12	Mid	Bünning (1973, Fig. 126)
*Pseudopidorus fasciasta*	1p	24, 60, 9	(3)	Li et al. (2003, Fig. 2)
*Pseudopidorus fasciasta*	1p	24, 60, 9	(3)	Wei et al. (2001, Fig. 4)
*Pteronemobius fascipes*	1p	24, 10, 8	(5)	Masaki (1989, Fig. 6)
*Pteronemobius fascipes*	1p	24, 60, 10	(3)	Masaki (1989, Fig. 2)
*Pterostichus nigrita*	2p,2°	24, 120, 8	Gulf Cos	Thiele (1977, Fig. 1A)
*Sarcophaga similis*	4p,11°	24, 120, 12	Mid (15)	Yamaguchi and Goto (2019, Figs. 4,5)
*Tetranycus urticae*	1p	24, 60, 10	Gulf & NC Cos (10)	Vaz Nunes and Veerman (1984, Fig. 2B)
*Thyrassua penangae*	1p	24, 45, 9	NC Cos (12)	He et al. (2009, Fig. 6C)
*Aphis fabiae*	1p	44, 60, 12	NW NE	Hardie (1987, Fig 3)
*Aedes atropalpus*	1p	48, 60, 12	NC Mtn	Beach and Craig (1977, Fig. 2)
*Chilo suppressalis*	1p	48, 60, 12	(12)	Chen et al. (2011, Fig. 6)
*Colaphellus bowringi *	1p	48, 60, 12	(7)	Wang et al. (2004, Fig. 8)
*Drosophila triauraria*	1p	48, 60, 10	(7)	Yoshida and Kimura (1993, Fig 3A)
*Mamestra brassicae*	1p	48, 60, 12	NW NE	Masaki (2001, Fig. 4)
*Megoura viciae*	1p	48, 60, 8	Mid	Lees (1973, Fig. 5I)
*Nasonia vitripennis*	1p	48, 60, 12	Mid & NC Cos	Saunders (1970, Fig. 1)
*Ostrina furnacalis*	1p	48, 60, 12	(8)	Yang et al. (2014, Fig. 7A)
*Ostrinia nubilalis*	1p	48, 60, 12	(11)	Bonnemaison (1978, Fig. 11)
*Pieris brassicae*	1p	48, 30, 12	NC Mtn	Bünning (1973, Fig. 126)
*Plodia interpunctata*	1p	48, 120, 12	(7)	Kikukawa et al. (2009, Fig. 3)
*Pseudopidorus fasciasta*	1p	48, 60, 12	NC Cos	Li et al. (2003, Fig. 7A)
*Pseudopidorus fasciasta*	1p	48, 60, 12	Mid	Wei et al. (2001, Fig. 8A)
*Chilo suppressalis*	1p	72, 60, 12	(7)	Chen et al. (2011, Fig. 6)
*Colaphellus bowringi *	1p	72, 120, 12	(7)	Wang et al. (2004, Fig. 8)
*Drosophila triauraria*	1p	72, 60, 10	(7)	Yoshida and Kimura (1993, Fig 3C)
*Mamestra brassicae*	1p	72, 120, 12	(14)	Kimura and Masaki (1993, Fig. 2)
*Megoura viciae*	1p	72, 60, 8	(14)	Lees (1973, Fig. 5O)
*Nasonia vitripennis*	1p	72, 120, 12	Gulf & NC Cos	Saunders (1970, Fig. 2)
*Ostrina furnacalis*	1p	72, 60, 12	(7)	Yang et al. (2014, Fig. 7C)
*Ostrinia nubilalis*	1p	72, 120, 12	(2)	Bonnemaison (1978, Fig. 11)
*Plodia interpunctata*	1p	72, 120, 12	(7)	Kikukawa et al. (2009, Fig. 3)
*Pseudopidorus fasciasta*	1p	72, 60, 12	NC Mtn	Li et al. (2003, Fig. 7C)
*Pseudopidorus fasciasta*	1p	72, 60, 12	(14)	Wei et al. (2001, Fig. 8C)
*Pteronemobius nigrofasciatus*	1p	72, 84, 11.5	(11)	Masaki (1984, Fig. 4)
*Sarcophaga argyrostoma*	1p	72, 60, 12	Gulf & NC Cos	Saunders (2002, Fig. 11.5)

**Headers**: **Pop,°N**, number of populations & latitudinal range if Pop > 1; **T,P,L: T** = L + D, h;** P** = duration of the light pulse, min; **L** = duration of the main photophase, h; ***Wy*(note)**, corresponding figure in *W. smithii* or (see Notes). **(2)** four peaks, 6-14 h apart. **(3)** one broad peak in mid-dark. **(4)** major peak at 16 h, minor peak at 58 h. **(5)** single peak late in dark. **(6)** two peaks with shallow valley in between them. **(7)** no apparent rhythmic trend. **(8)** single peak early dark; high long-day response rest of dark. **(9)** no late dark light pulse. **(10)** peak in middle of dark; no response early or late dark. **(11)** three peaks, early, mid, & late dark. **(12)** narrow A peak & broad B peak. **(13)** after reared in LL or L:D = 10:14, broad mid-dark peak or broad A peak only; no difference between north & south over 9°latitude. **(14)** Single peak early in dark. **(15)** late-dark peak declines with increasing latitude

### Mountain vs. lowland populations

The NC mountain populations share a common photic environment with NC coastal populations at the same latitude, but a more northern seasonal environment. Consistently, the independently derived Pocono Mountain population shares a similar photic environment with the nearby lowland mid-latitude populations, but a more northern seasonal environment. Response to both resonance experiments (Bradshaw et al. 2003) and ASPPs (Figs. 3, 4, 5) by the mountain populations resembles northern populations underscores that seasonality, not summer day length, has selected for decreased response to both protocols.

### Response to 72 h cycles

None of the response profiles in 24 h or 48 h cycles provides evidence for a circadian basis of photoperiodic response. All peaks in response to light breaks occur within 24 h of the main, 10 h light period. Hence, either a circadian or an interval (hourglass) timer provides an equally plausible interpretation of the data. Evidence for a circadian basis of photoperiodic time measurement requires the occurrence of a peak response in the middle of a much longer dark period (Saunders 1970, p. 603): “The 72 h experiment also removes the objection that each peak represents the result of a direct interaction between the pulse and the main light component. In other words, the middle peak is too far removed from either the preceding or following light periods to be caused by a direct interaction,” i.e., by an interval timer. With *T* = 72 h, the middle peaks in Fig. 5e-f unambiguously occur more than 24 h after dusk and more than 24 h before dawn of the main light period. They can plausibly be attributed to a self-sustaining, rhythmic process underlying photoperiodic time measurement in those populations.

The same southern populations in 72 h ASPP cycles (Fig. 5e-f) also show a strong, rhythmic response to resonance (Nanda-Hamner) experiments (Wegis et al. 1997; Bradshaw et al. 2003). In *W. smithii*, the circadian prediction is upheld in the 30–35°N, southern lowlands by a strong biphasic response (Bradshaw et al. 2003), consistent with the mid-dark peak in response to 72 h ASPPs. Using the mid-dark intervals to estimate the period of circadian oscillation related to photoperiodic response indicates τ ranging from 20.0 to 22.5 h (Table 3). Hence, both resonance experiments and ASPPs in 72 h cycles support a functional connection between photoperiodic response and a circadian oscillator with *τ* < 24 h in southern populations of *W. smithii*.

**Table 3:** Circadian period (*τ*) estimated from response to resonance experiments and ASPPs

Population in Table 1	Region	Resonance: peak-to-peak interval (h)^a^	ASPP T = 72 h: valley-to-valley interval (h)^b^	ASPP T = 48 “C” peak h after dawn^c^	ASPP T = 48 “C” peak h before dusk^c^
●	Carolina Coast	20	22.5	21	23
◯		20	20.0	21	23
●	Gulf Coast	20	22.5	21	21
		20	22.5	21	21

^a^Bradshaw et al. (2003, Fig. 4)

^b^Fig. 5

^c^Fig. 4

We now interpret the “C” peaks in 48 h cycles (Fig. 4e–f) in the light of the above conclusion. The “C” peak in the early dark occurs consistently 21 h after the preceding dawn; the “C” peak in the late dark occurs 21–23 h before the following dusk (Table 3). Hence, the position of both “C” peaks temptingly connects photoperiodism in *W. smithii* with an underlying circadian oscillator, consistently with *τ* < 24 h. Again, the caveat here is that the relationship of the “C” peaks could be explained by the output of an interval (hourglass) timer as well as by the output of a circadian oscillator, since each occurs less than 24 h within a dawn or dusk transition. However tempting this connection might be, “It is only when T is extended far beyond 24 h that the clearly circadian nature of the photoperiodic timer is revealed, with the interpeak interval showing the period *τ* for that part of the circadian system” (Saunders 2004, p.9). Importantly, this “circadian nature” resides only within the southern and not the more evolutionarily derived northern and mountain populations (Fig. 5; Wegis et al. 1997; Bradshaw et al. 2003).

If we conclude that a circadian oscillator underlies photoperiodism in the southern populations, then we also have to conclude that something else is responsible in more evolutionarily derived northern and mountain populations. Pittendrigh and Minis (1971, p. 243) recognized this dichotomy:

“In general, resonance experiments are (1) always powerful when a positive resonance effect is found; but (2) powerful when one is missing only if the system remains for a sufficiently long time in the inducible state for the resonance effect to occur. The happy circumstance that [when] escape from diapause… is under photoperiodic control does, however, leave open the possibility of performing more meaningful resonance experiments... The long-enduring steady-state afforded by diapause, lasting for weeks, will permit us to subject the system to a nearly unlimited number of cycles.”

Even though Pittendrigh never capitalized on this insight, we were able to run our 72-h ASPP and resonance experiments for 10 weeks with a long-day L:D = 18:54 control, exploiting Pittendrigh’s “happy circumstance” and showing that the duration of our experiments was sufficiently “powerful” to elicit a long-day response, if it were physiologically functional. If we accept the hypothesis that circadian rhythms underlie photoperiodism based on “positive” responses to resonance or long ASPP experiments, then we must conclude that something else is functionally responsible for “negative” responses; otherwise, we are testing a non-falsifiable hypothesis. Emerson et al. (2009) conducted a resonance experiment with a long-day main light period of 18 h. In this case, > 95% of replicated southern, northern, NC coastal, and NC mountain populations all exhibited a long-day response regardless of night length; *W. smithii* evaluates day-length, not night length, as previously concluded from independent experiments (Bradshaw et al. 1998). In addition, development times by both coastal and mountain populations were a linear function of *T*; but in both southern and northern populations were a rhythmic function of *T* with *τ* < 24 h [lowland, 23.9 ± 1.6 h; mountain, 22.3 ± 1.1 h (± 2SE)]. Hence, there was little correspondence between a measure of circadian rhythmicity (development time, as in Saunders 1972) and rhythmic response to short-day resonance or ASPP experiments.

Response to resonance experiments in *W. smithii* is not 100% and selection experiments show that there is heritable (genetic) variation for both positive and negative responses within populations (Bradshaw et al 2003, 2005; Mathias et al. 2006). The conundrum, then, is how insects, like *W. smithii* and Pittendrigh’s *Pectinophora,* that do not show a “positive” resonant effect (but possess the genetic capacity to do so) or do not show a rhythmic response to long-cycle ASSPs, are nonetheless still able to measure day or night length. What then is the role of the circadian clock in the photoperiodic timer of individuals in the same species, or even in the same population that do not respond to resonance experiments or to ASPPs in 72 h cycles (Fig. 5; Table 2, footnote 7)? The answer to this question depends in large part upon whether the investigator is focused primarily on establishing the mechanistic connection between the circadian clock (clock) and the photoperiodic timer (timer), typically in a single population (Table 2), or all too rarely is focused on how that connection varies geographically or through evolutionary time.

### Mechanisms underlying photoperiodic time measurement

The results in Fig. 5e, f provide firm support for a circadian-connected mechanism underlying photoperiodic response in the broader southern clade of *W. smithii*. Results involving the broader, evolutionarily derived northern clade (Fig. 5a–d) are less definitive: some populations show a low peak in the circadian-expected mid-dark; other populations do not. This observation prompts the question: what mechanisms are responsible for the negative as well as the positive responses?

First, non-response to 72 h ASPPs in northern populations could be due to an increasing reliance on an interval (hourglass) timer in more recently derived populations (Bradshaw and Holzapfel 2001b; Merz et al. 2013). Second, non-response could be due to light pulses occurring at times from dawn or dusk that exceed the critical photoperiod (Bradshaw et al. 1998); however, this explanation is consistent with either a circadian sensitivity rhythm or an interval timer. Third, reduced response could be due to a rapidly damping oscillator (Saunders et al. 2004), but see Damped oscillator, below. Fourth, the photoreceptor-transmission system may simply be less sensitive to light in northern populations. However, quantum-specific action spectra in a northern population between Mid and NE show that dawn and dusk transitions respond to light at similar wavelengths and intensities at or below that of the full moon (Bradshaw and Phillips 1980), far less bright than experienced in the ASPP experimental chambers.

Finally, populations in the broader northern clade (Fig. 2) require more long days than populations in the southern clade to terminate diapause (Bradshaw and Lounibos 1977). The low mid-dark responses of populations in Fig. 5a–d might then be ascribed to an insufficient number of inductive cycles to trigger a greater long-day response. However, there are sufficient cycles to induce a > 90% long-day response in the early and late subjective night in the Mid population (Fig. 5d) that shares a most recent common ancestor with the northern and NC Mtn populations. Hence, the low, but definitely present mid-dark response in the Mid population cannot be ascribed to insufficient inductive cycles. More likely, the Mid population is polymorphic for an underlying circadian-connected and an alternative mechanism. Following this same reasoning, the relative roles of circadian vs. non-rhythmic mechanisms remain ambiguous and unresolved in the evolutionarily derived northern NW and NE populations, while non-circadian mechanisms predominate in the independently derived NC mountain populations. These results underscore the importance of taking into account the required number of cycles to induce a long-day response among populations when interpreting the results of ASPP or resonance experiments. When so doing, these results also show that within the species *W. smithii*, the most likely mechanisms underlying photoperiodic response range from firmly circadian, to polymorphic, to ambiguous, to firmly arrhythmic.

Comparisons among populations of *W. smithii* demonstrate the diversity of physiological mechanisms underlying photoperiodic response generated by natural selection within a single species. No single population, geographic region, or phylogenetic clade alone can be identified as representative of photoperiodism in an entire species; yet, documented examples of ASPPs among other species of insects and mites are overwhelmingly represented by a single population (Table 2). Variable responses to ASPPs in *W. smithii* illustrate not only the power, but also the necessity of considering multiple populations of known phylogenetic relationship in varying seasonal contexts when evaluating mechanisms underlying photoperiodism within as well as among species.

Below, we address the question of the relationship between the circadian clock and the photoperiodic timer in the context of more comprehensive models.

### Quantitative photoinducible phase, φi

“Insects synthesize the hypothetical diapause inducing substance based on how long the φi is exposed to light. The synthetic rate of the substance is higher during shorter days but lower during longer days. The substance accumulates in the counter system. When the level of the accumulated substance exceeds a certain ‘internal threshold,’ diapause induction is determined, whereas nondiapause development is determined when the accumulation is lower than the threshold…” (Yamaguchi and Goto 2019, pp. 294 & 296). Yamaguchi and Goto (2019) only applied their model to 24 h cycles, so their results with *Sarcophaga similis*, as in *W. smithii* (Fig. 3d), are equally compatible with either an interval (hourglass) or a circadian timer.

### Damped oscillator

A damping oscillator is one whose amplitude declines through time or with increasing cycles, generally without altering its period. Although invoked to explain non-response (negative) to longer cycles such as observed in resonance or longer ASPP cycles (Fig. 5a–c), a sufficiently damped oscillator would also account for shorter cycles as well. A damped oscillator was suggested by Bünning (1973, p. 215) as a complicating factor in experiments with extended dark. This concept was refined and elaborated upon by Saunders and colleagues in a series of models (Lewis and Saunders 1987; Saunders and Lewis 1987a, b, 1988). However, qPCR of RNA from heads of larval *W. smithii*, expression of the core circadian clock gene, period, does not vary in period, amplitude, or damping between coastal and mountain North Carolina populations (Bradshaw and Holzapfel 2017, Fig. 4) that clearly differ in critical photoperiod (Table 1) and response to ASPPs (Figs. 3, 4, 5) or resonance experiments (Bradshaw et al 2003). Results from qPCR of *timeless*, *Clock*,* cycle*, and* cry-2* are currently being analyzed.

### Circadian amplitude

Pittendrigh et al.’s (1991) “amplitude hypothesis” incorporated geographic variation, i.e., evolution, into consideration. They proposed that a “dependence of [circadian] pacemaker amplitude on photoperiod and temperature underlies the cell’s measurement of daylength” (p. 312) and accounts for latitudinal clines in photoperiodic response. Pittendrigh et al. (1991) developed their model to account for the same topological patterns as Saunders and Lewis (Lewis and Saunders 1987; Saunders and Lewis 1987a,b). Both models were strictly theoretical and based on ad hoc parameterization.

Pittendrigh et al.’s model (1991, Fig. 9) did specifically lend itself to the prediction that geographic variation in photoperiodic response results from a vertical shift in the entire photoperiodic response curve, not only at ecologically relevant day lengths, but also at the extremes not encountered by natural populations at temperate latitudes (Pittendrigh and Takamura 1987). This prediction was borne out in *W. smithii* (Wegis et al. 1997; Fig. 3; Bradshaw et al. 2003; Fig. 3). What we don’t know is whether the prediction was upheld due to evolutionary modification of the circadian clock, the downstream photoperiodic timer, clock-timer connectivity, or a (epistatic) combination thereof.

Importantly, Pittendrigh and Takamura (1993) later selected on critical photoperiod and, separately, on adult eclosion rhythmicity in *D. Auraria* and found (their Fig. 11) that “Major differences between strains established [in nature] or by laboratory selection in their photoperiodic responses…are not matched by comparable differences in circadian rhythmicity of their eclosion activity.” “An even greater difference between the Early and Late [laboratory-selected] strains in their eclosion rhythmicity… is similarly unmatched by any change in photoperiodic response,” from which they invoked an additional (slave?) oscillator, and concluded “The pacemakers responsible for the eclosion rhythm and the photoperiodic response are different.” This conclusion underscores that no matter how tantalizingly delicious the resemblance, parallels between overt behavioral rhythms and photoperiodism are a miasmatic swamp, highly prone to misdirection.

### Commensal model

Given that organisms live in a perpetual 24 h world with sunrise and sunset from about 60°S to 60°N (US Naval Observatory, 2022), maintaining daily temporal programming at temperate latitudes will be under strong stabilizing selection. Selection on seasonal temporal programming will be stabilizing at the local scale but subject to directional selection over a geographic scale or during episodes of climate change. The goal, then, is to propose a way in which circadian-connected photoperiodic response can evolve without perturbing daily temporal programming (Bradshaw and Holzapfel 2010a). We call it a “commensal model” (Bradshaw and Holzapfel 2017, Sec. 4.3, Denlinger et al. 2017, Sec. 6c) after the ecological term, *commensalism*, a symbiotic relationship in which one partner benefits (evolution of seasonal timing) without affecting performance of the other partner (regulation of daily timing).

**Fig. 6:**
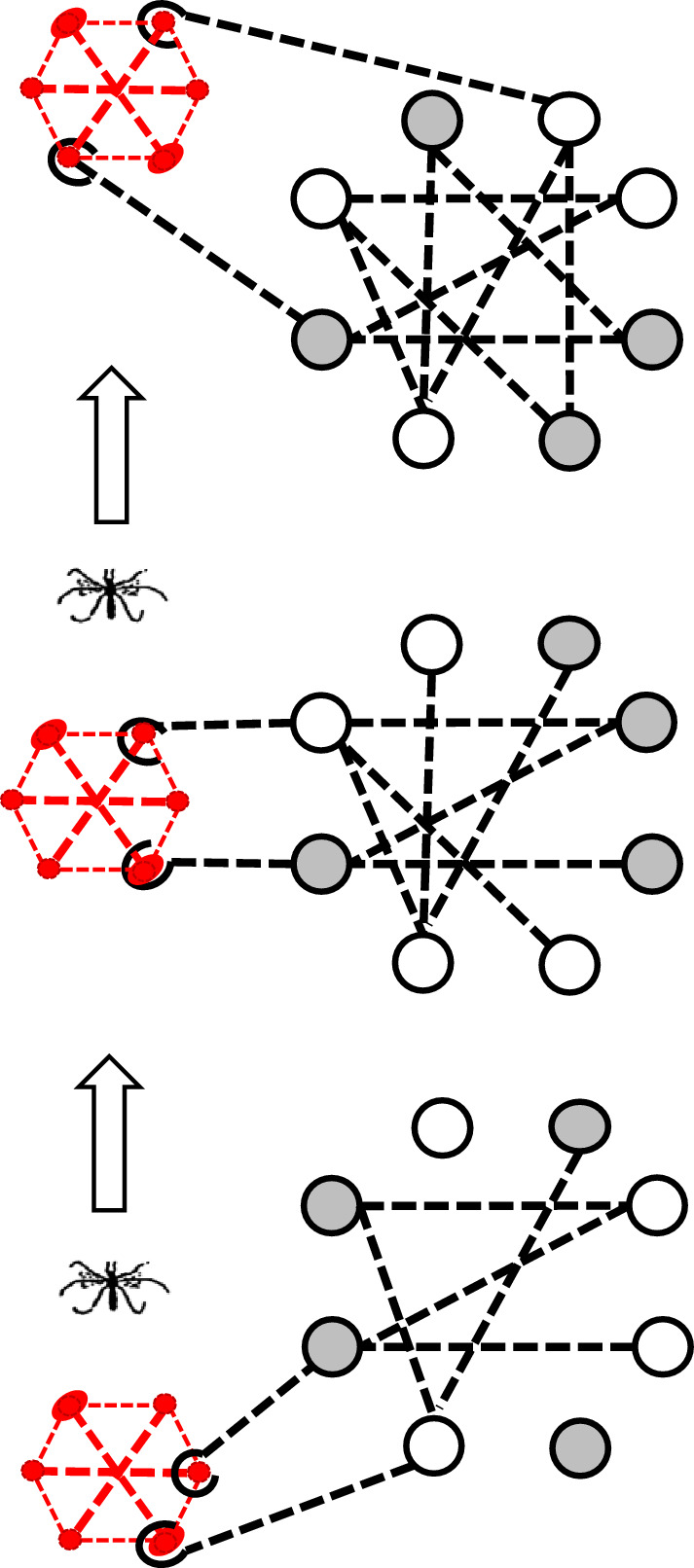
Commensal model for the evolution of photoperiodic time measurement (timer, black) and its connection with a circadian pacemaker (clock, red) using *Wyeomyia smithii* as an example. The clock remains constant; the clock-timer connection and the timer evolve. White circles, independent (additive) allelic effects; gray circles, interaction between alleles (dominance); dashed lines, gene–gene interaction (epistasis). Arrows indicate direction in evolution. Black hooks in the clock-timer connection indicate one-way interaction between the clock and timer; in a sense, the timer “captures” time information from the clock without perturbing circadian daily organization. Internal connections within the photoperiodic timer reflect the fact that genetic variation (additive genetic variance) for critical photoperiod increases in more derived populations (Hard et al. 1993). Heritable variation in epistasis occurs within a population (Bradshaw et al. 2005) and among southern, northern, and mountain populations (Hard et al. 1993; Lair et al. 1997)

As *W. smithii* has expanded its range progressively from the southeastern coastal plain to more northern latitudes and higher elevations (Fig. 2), a few founding individuals will each time enter a wide-open, competition-free habitat of already established pitcher plants, generating a founder-flush episode (Carson 1968) of successive genetic drift, population growth, and selection. Each episode can generate “an expanding web of pleiotropic effects and epistatic interactions” (Templeton 2000, p 48), resulting in “genetic revolutions” (Mayr 1963, p. 533) throughout the genome, including the photoperiodic timer (Hard et al. 1993; Bradshaw and Holzapfel 2000, 2001b). Evolution of photoperiodic response can then evolve independently of circadian clock function, as a result of genetic “revolutions” in the downstream photoperiodic timer itself, and clock-timer connections (Fig. 6).

From this model, we can make the principal prediction: there are alleles segregating in natural populations that affect photoperiodic response (critical photoperiod) that do not alter clock function; likewise, there are circadian clock gene alleles segregating in natural populations that affect clock performance but not photoperiodic response. An example of the latter in European *Drosophila melanogaster* is the *s-tim* and *ls-tim* polymorphism that affects clock performance, and pleiotropically, the incidence of diapause (Tauber et al. 2007; Sandrelli et al. 2007), development time, and early fecundity (Andreatta et al. 2023), but not photoperiod x clock genotype interaction (Tauber et al. 2007, Fig. 3). We predict that other such examples will emerge as more is known about allelic variation in photoperiodic response genes and in clock genes segregating in natural populations of robustly photoperiodic arthropods.

### Geography, genetics, and evolution

Since overt behavioral rhythms are unreliable indicators of circadian-photoperiodic connectivity (Pittendrigh and Takamura 1993), we propose making inferences about clock-timer evolution over geographic gradients following the protocol in Box 2. Hypotheses and models are only as good as their ability to make a priori predictions that are testable and realistically falsifiable in the crucible of a posteriori experimentation. In short, establishing an evolutionary connection between the circadian clock and the photoperiodic timer ultimately, and necessarily involves testing whether or not there is a hard genetic connection between them, not just parallel peculiarities.

Box 2. Testing for a causal role of the circadian clock (CC) in the evolution of photoperiodic time measurement (PTM) over eco-climatic gradients [CC measured by clock gene sequence, clock gene rhythmic expression, or rhythmic response of critical photoperiod to resonance experiments or to ASSPs with T ≥ 72 h]. #1. Is there a correlation between PTM & CC behavior over a geographic gradient?

No: the clock and timer have evolved independently^1^

Yes: go to #2

#2. Are the residuals from the separate regressions of PTM & CC on geography correlated?

No: the geographic correlation likely due to parallel evolution, not a causal relationship^2^

Yes: CC possibly involved in evolution of PTM; go to #3

#3. Does selection on PTM elicit a correlated response in CC behavior or vice versa

No: genetic variation in the CC not likely involved in the evolution of PTM^3^

Yes: genetic variation in the CC possibly involved in the evolution of PTM; go to #4^4^

#4. Does antagonistic selection separate or reverse the genetic correlation between the CC and PTM?

No: genetic variation in the CC is a causal factor in the evolution of PTM over a geographic gradient.

Yes: the clock and timer can evolve independently^5^

Examples: ^1^Vaz Nunes et al. 1990; ^2^O’Brien et al. 2011; ^3^Pittendrigh and Takamura 1993, Fig. 11; ^4^Bradshaw et al. 2003; ^5^Lankinen and Forsman, 2006; Bradshaw et al. 2012.

### Micro- and macro-evolution

We define micro-evolution as evolution taking place within and among populations of a single species; macro-evolution is evolution taking place between different species or higher taxa. In his review of insect photoperiodism-circadian gene interaction, Goto (2022, p. 203, and his Table 2), describes “Discrepancies of the roles of clock genes in photoperiodism.” In brief, Goto documents variation in the effect of circadian gene-knockdowns in typically a single population of different species of insects. If we examine the pattern of response to ASPPs across diverse species of insects and mites (Table 2), most of the patterns among seven orders of arthropods are represented among populations within *W. smithii* alone (Figs. 3, 4, 5). Through the lens of evolutionary time in *W. smithii*, we do not find these “discrepancies” to be surprising.

Whatever the connections between the circadian clock and the photoperiodic timer, that connection is itself variable among individuals within a population and among populations, as well as between species or higher taxa. Micro-evolutionary patterns clearly illustrated among geographically distinct populations of *W. smithii* provide an example of the sort of micro-evolution within a species that, over time and space, could lead to macro-evolutionary variation in genetic connections between the daily circadian clock and the seasonal photoperiodic timer among species and higher taxa.

We have proposed that the connection between the circadian clock and photoperiodic time measurement is genetically and evolutionarily flexible due to a pleiotropic relationship between individual clock genes and the photoperiodic timer (Fig. 6). This flexibility would conserve daily time keeping by the circadian clock in a consistent 24-h world and still permit rapid evolutionary flexibility of the photoperiodic timer when populations are confronted with seasonal selection that varies in geographic space during range expansion (Cooke 1977; Stiling 1993; Saikkonen et al. 2012; Urbanski et al. 2012; Lehman et al. 2014; Armbruster 2016) and that varies in time during periods of rapid climate change (Bradshaw and Holzapfel 2001a, 2008, 2010b).

## Conclusion

The motivation for our experiments has been, from the outset (Bradshaw 1976; Bradshaw and Lounibos 1977), to determine the physiological and genetic basis for the evolution of photoperiodic time measurement over the wide geographical and climatic gradient of eastern North America. In considering geographic variation, we are, at the same time, looking at the endpoints of evolution, that is, genetic as well as phenotypic consequences of natural selection in seasonal environments. This perspective has typically been viewed on a macro-evolutionary scale, using comparisons among species or higher taxa; but, macro-evolution originates from heritable variation within and among populations of single species. In *W. smithii*, we are able to place physiological variation of photoperiodic response not only in a geographic, but also in a phylogenetic context. Northeastern, northwestern, and mountain populations all share a most recent common ancestor with mid-latitude populations, which themselves share progressively more remote common ancestors with Carolina coastal and Gulf coastal populations. A walk across geographic space is also a journey through evolutionary time.

Understanding genetic variation among different species originates from studying genetic variation within and among populations of diverse higher taxa. Importantly, an example of such genetic variation exists within and among populations of *W. smithii*. Given the different selection pressures on the circadian clock and the photoperiodic timer, the connection between them is to be expected to vary at both the molecular and population-genetic levels, leading to the genetic variation we have documented within a single species, as well as the molecular discrepancies among species. Only determining variation between the clock and timer over multiple, broad geographical distances can one understand the physiology, genetics, and evolution within even a single species. Then, and only then, do comparisons among multiple species lead to robust macro-evolutionary conclusions.

We have shown experimentally that the clock and timer can and have evolved independently. Nonetheless, we conclude that the clock and timer can somehow be connected, but the way they are connected differs among populations, species, and higher taxa. The crucial question remains as to how that somehow varies over evolutionary time across levels of biological integration from individuals to species and higher taxa. “Nothing makes sense in biology except in the light of evolution” (Dobzhansky 1964, p. 449).

### Supplementary Information

Below is the link to the electronic supplementary material.

Supplementary file1 (XLSX 17 KB)

## Data Availability

Original data for Figs. 3, 4, 5 may be found as Excel spreadsheets in the supplemental data files.

## Footnotes

1 “…dass die physiologische Grundlage des eben gennanten Photoperiodismus in dieser endonomen Tagesrhythmik liegt.”.

## Glossary

ASPP: Asymmetric Skeleton PhotoPeriod (aka Bünsow protocol) consisting of one longer and one shorter light pulse per L:D:L:D cycle
Circadian: An internal, temperature-compensated, self-sustaining rhythm with a non-zero amplitude and a non-zero period of oscillation (τ) of about 24h
Commensal: A symbiotic relationship in which one partner benefits without cost or benefit to the other partner. Symbiosis means simply “living together. Correlated response to selection—see Box 1
CPP: Critical PhotoPeriod, the 50% intercept on the photoperiod axis of a PPRC
Dominance: Interaction between alleles at the same locus
Epistasis: Interaction between alleles at different loci, aka gene-gene interaction
Evolution: A genetic change in a population through time
Genetic correlation: Correlation between two traits due to genetic effects, exclusive of environmental effects—see Box 1
GXE: Genotype by environment interaction, where the effects of a gene on the phenotype vary depending on environmental context; genetic variation in critical photoperiod being a relevant example
L:D: Hours light:dark
Master vs. slave: The driving vs. a driven oscillator
Oscillator: A repeating function with a non-zero amplitude and a non-zero repeat period
Pacemaker: An oscillator that drives other oscillators
Phenotypic correlation: Correlation between traits due to combined genetic plus environmental effects
Photoperiod: Duration of the light in an L:D cycle
Photoperiodic counter: Herein, accumulation of the number of long days sufficient to induce development
Photoperiodism: Response of some trait to day or night length, usually in a seasonal context
Photophase: Light portion of an L:D cycle = photoperiod
Pleiotropy: Effect of a gene or group of genes affecting multiple traits
PPRC: Photoperiodic Response Curve = plot of expression of a trait as a function of photoperiod, usually sigmoid in shape
Resonance experiments: A short day followed in separate treatments by a series of long nights, generally varying T = 10+D from 24-72h (aka Nanda-Hamner protocol)
Response to selection: Genetic change in a population after selection has been imposed on it: R =h^2^S, where h^2^ is the heritability of the trait and S is the selection differential
Rhythm = Oscillation: A repeating function with both a non-zero amplitude and a non-zero repeat period
Scotophase: Dark portion of an L:D cycle
Selection: Artificial, investigator determines reproductive success based on a specific trait or traits; natural, environment (laboratory or nature) determines reproductive success
T: Period of an external cycle, usually light+dark
τ: tau, duration of an internal unrestrained (free running) circadian cycle
